# Sepsis in neonates: experience in a tertiary-care hospital

**DOI:** 10.1186/cc11745

**Published:** 2012-11-14

**Authors:** I Guerrero-Lozano, A Alonso-Ojembarrena, F Galán-Sanchez, AM García-Tapia, P Marin-Casanova, P García-Martos, M Rodriguez-Iglesias

**Affiliations:** 1 Puerta del Mar University Hospital, Cadiz, Spain

## Background

Sepsis is a common condition in newborns, which has significant morbidity and mortality worldwide. In recent years, multiple factors have led to an increase in its incidence, such as the use of invasive diagnostic procedures, broad-spectrum antimicrobial therapy and an increase of immunocompromised patients. There have also been changes in the profile of the agents causing sepsis. The aims of this study were the determination of the annual incidence of neonatal sepsis (early-onset and late-onset), analyzing their clinical course, significant microorganisms isolated and the profile of antimicrobial resistance.

## Methods

One hundred and ninety-eight cases of sepsis were studied, collected over the past five years (2007 to 2011). Early-onset neonatal sepsis was defined as that which appeared before 3 days of life and late-onset neonatal sepsis if it happened later. Blood cultures were incubated in the BACTEC FX System (Becton Dickinson), and identification and antimicrobial susceptibility testing were done by the Wider system (Soria Melguizo). Yeasts were identified by ID32C (bioMerieux).

## Results

Of the 198 detected sepsis cases, 173 (87.4%) were late and 25 (12.6%) were early. While the annual incidence rate of early-onset sepsis was uniform in each year, the late-onset sepsis has experienced an increase in 2011. The mortality rate was similar throughout the study (10.8%). Gram-negative bacilli are the most frequently isolated (48%), with predominance of enterobacteria compared with nonfermenters (84 vs. 11 cases), followed by Gram-positive cocci (36%) and yeast (16%). In candidemia most common are nonalbicans species of *Candida *(27 vs. 5 cases), with *Candida parapsilosis *more frequently isolated (65.6%). *Klebsiella pneumoniae *(33 strains) was the more frequent microorganism, followed by *Escherichia coli*, *Staphylococcus epidermidis*, *C. parapsilosis *and *Enterobacter cloacae*. With respect to antibiotic resistance, 11.1% of *Staphylococcus aureus *were MRSA and 19% (16 strains) of enterobacteria were ESBL producing. Only one strain of *Pseudomonas aeruginosa *was resistant to imipenem by metallobetalactamases.

## Conclusion

*K. pneumoniae *was the organism responsible for more episodes of sepsis in neonates. As in other studies in neonatology, this study highlights the prevalence of nonalbicans species in candidemia due to the frequency of *C. parapsilosis*. Late-onset sepsis has increased by Gram-negative bacilli in the last year, probably due to the occurrence of a nosocomial outbreak of ESBL-producing *E. cloacae*. However, the mortality rate from sepsis has remained stable. When clinically evaluated, cases of sepsis can be detected as a predominance of enterobacteria against *S. epidermidis*, highlighting the importance of careful analysis of clinical outcomes.

